# Failure Rate of Pediatric Dental Treatment under General Anesthesia

**DOI:** 10.3390/dj6030025

**Published:** 2018-06-22

**Authors:** Effat Khodadadi, Mehrnaz Mohammadpour, Saeed Reza Motamedian, Farnaz Kouhestani

**Affiliations:** 1 Department of Pediatric Dentistry, Faculty of Dentistry, Babol University of Medical Sciences, Babol 47176-47745, Iran; efat.khodadadi@gmail.com; 2 Department of Pediatric Dentistry, Faculty of Dentistry, Shahrekord University of Medical Sciences, Shahrekord 81639-48111, Iran; mehrnaz_dnt@yahoo.com; 3 Department of Orthodontics, School of Dentistry, Shahid Beheshti University of Medical Sciences, Tehran 19839-63113, Iran; sr.motamedian@yahoo.com; 4 Department of Periodontics, School of Dentistry, Shahid Beheshti University of Medical Sciences, Tehran 19839-63113, Iran

**Keywords:** composite resins, dental amalgam, dental pulp diseases, general anesthesia, pediatric dentistry, treatment failure

## Abstract

**Aim:** To assess the failure rates of various pediatric dental treatments performed under general anesthesia (GA) after six months to five years of follow-up. **Design:** This multicenter retrospective cohort study was performed on patients treated by five pedodontists in two private hospitals located in northern Iran during 2010–2013 and comprised 155 patients. The patients were recalled and clinically examined. During the clinical examination of the primary teeth, oral hygiene, dmft index, and failure of previous treatments was evaluated. The data were analyzed using the Chi square and regression analyses with a significance level of 0.05. **Results:** 114 patients (74 males and 40 females, mean age: 37.17 ± 10.75 months) with 1155 primary teeth treated under GA participated in the follow-up. The overall failure rate was 6.59%. The failure rates of pulpectomy, pulopotomy, fissure sealant, stainless steel crown (SSC), amalgam, and composite fillings were 2.90%, 3.03%, 4.83%, 5.26%, 5.33%, and 9.63%, respectively. Among the confounding factors, only gender had a significant effect on the anterior composite failure rate (*p* = 0.029) and age had a significant effect on the failure rate of fissure sealant therapy (*p* = 0.015) and SSC (*p* = 0.018). **Conclusion:** The overall rate of treatment failure in pediatric patients, treated under GA, was 6.59%.

## 1. Introduction

Although many pediatric patients receive dental treatment by simple behavior control techniques like “Tell-Show-Do”, some uncooperative patients with extensive dental caries and need of several treatment sessions should undergo general anesthesia (GA) for effective treatment [1,2]. It could provide more efficient dental treatment in a safe environment in one session with minimum mental and physical anxiety [3,4]. The most common dental treatments for children, under GA, include restorative treatments, pulp therapy, minor surgeries, and extractions [5]. As dental and oral diseases are treated in one session under GA, an immediate improvement in oral health-related quality of life both for the patients and their families is observed [6].

Although dental treatments under GA provide relatively easier access for the clinicians, they are costlier for the parents. Some authors have stated that GA might not be cost-effective as there might be a higher failure rate and higher incidence of secondary caries in children who are treated under GA [7,8]. Hence, it is of great importance to assess the long-term prognosis of these treatments. Previously, few reports have evaluated the outcome of individual treatments of dental procedures done under GA [2,9,10]. Their results indicated high success rates of stainless steel crowns (SSC) and amalgam fillings compared to composite fillings as well as a high success rate of pulpotomy. However, other studies reported high failure rates of SSC [11,12] and pulpotomy [11]. Also, the follow-up of these studies lasted for less than two years and their sample size was less than 80. Therefore, it is necessary to evaluate the failure rate of dental treatments done under GA in larger populations and with longer follow-up periods.

The current study was performed to assess the failure rates of various pediatric dental treatments performed under GA and their influencing factors, with follow-up from six months up to five years after treatment, in two private hospitals in northern Iran.

## 2. Results

### 2.1. Patients

The overall documents of 813 pediatric dental treatments performed under GA were evaluated, out of which 155 (19.1%) were considered eligible. These patients were contacted to participate in the follow-up sessions. Among these patients, 114 patients (73.5%) participated successfully in the follow-up sessions. Among these patients, 74 (64.9%) were males and 40 (35.1%) were females. The mean age of the patients at the time of GA was 37.17 ± 10.75 months (range: 16–65 months) and at the time of follow-up was 71.14 ± 18.37 months (range: 30–100 months). The mean follow-up time was 33.97 ± 15.60 months (range: 7–66 months). Only 58 patients (50.88%) had participated in fluoride therapy sessions after the treatment under GA.

The current oral health status of the patients is presented in Table 1. Oral hygiene, based on the simplified oral health index (OHI-S; modified), was moderate (mean = 2.67) and the dmft index was high (mean = 10.78), mostly due to the fillings.

Overall, 1254 primary teeth were treated under GA in the included patients. Among the treated teeth, 99 (5.85%) of them were lost due to exfoliation. These teeth received 13 fissure sealant, 47 anterior composite restoration, nine posterior composite restoration, 19 amalgam filling, 11 SSC, 14 pulpotomy, and nine pulpectomy treatments. As it was not possible to evaluate the failure of the treatments in the exfoliated teeth, they were excluded from the study. Therefore, only 1155 teeth, which received 1594 treatments, were included.

### 2.2. Failure Rate

The overall failure rate of the dental treatments performed under GA was 6.59% (105 failures). Table 2 depicts the failure rate of each treatment modality. Posterior composite restorations had the highest failure rate, whereas pulpectomy had the lowest. The failure rates of the posterior (9.96%) and anterior (9.44%) composite restorations were higher as compared to the other restorations. The difference between the posterior and anterior composite failure rates was not significant (*p* = 0.837, power = 5%). Although the posterior composite restorations had a higher failure rate (9.96%) as compared to the amalgam fillings (5.33%), the difference was not statistically significant (*p* = 0.112, power = 88%). In addition, the difference between the failure rates of pulpotomy (3.03%) and pulpectomy (2.90%) was not significant (*p* = 0.962, power = 3%).

**Figure 1:**
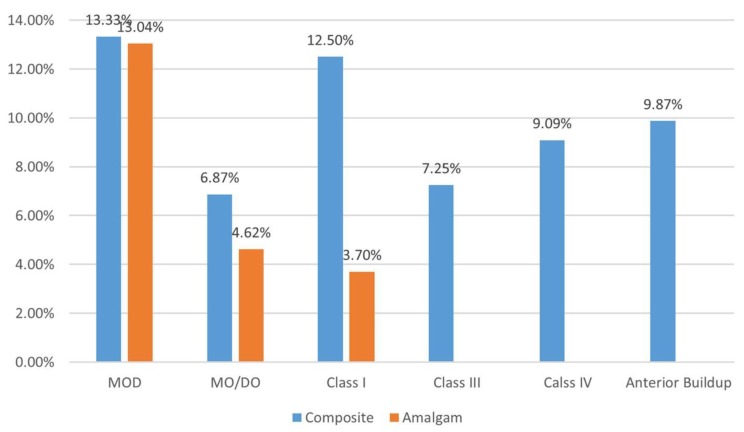
Comparison of failure rates of amalgam and composite restorations. Each bar shows the failure rate (ratio of failure/total treated teeth × 100) of that kind of restoration. The overall failure rate of amalgam restorations was 5.33% (9 out of 169) and for posterior composite restorations it was 9.96% (26 out of 261). No amalgam filling was done in Class III, IV, and anterior buildup.

Among the seven failed sealants of the primary molars, three (42.86%) needed a new sealant, whereas the other four needed restorative treatment. As demonstrated in Figure 1, the posterior composite restorations had a higher failure rate as compared to the amalgam restoration. The restorations that involved more tooth surfaces had higher failure rates as compared to those with lesser surfaces, except the single surface Class I composite fillings, which had 12.50% failure rate (Figure 1).

### 2.3. Influencing Factors

The effect of the confounding factors on the treatment failure rates was assessed using a regression model. It was found that only gender had a significant effect on the anterior composite failure rate (*β* = −7.436, *p* = 0.029). This means that boys had 7.436% less failure in the anterior composite failure as compared to girls, when considering the other confounding factors. In addition, age had a significant effect on the failure rates of the fissure sealant therapy (*β* = −0.453, *p* = 0.015) and SSC (*β* = 0.353, *p* = 0.018). This demonstrates that for every one-year increase in the patient’s age, the outcome variable (i.e., failure rate) increases by the beta coefficient value. Other factors, including the parents’ education, oral health, and dmft indices had no significant effect on the failure rates (*p* > 0.05).

The rate of each treatment failure, based on the time of follow-ups, indicated an increasing trend in regard to the total failures (Table 3); however, the regression model indicated no significant effect of the follow-up time on the failure rate (*p* = 0.604).

## 3. Discussion

The diet trend of children tends toward cariogenic foods, which increases the need for dental treatments. Since it is unavoidable, in the case of some children, to perform the dental treatment under GA, it is necessary to assess the success and failure of such treatments in order to improve the health care standards as well as avoid relapse [3,13,14]. The current study aimed to evaluate the failure rates of pediatric dental treatments performed under GA. The results indicated low failure rates of pulp therapy, fissure sealant, SSC, and amalgam restorations.

Previous studies have revealed the success rate of the fissure sealant therapy to be between 82% and 88.6% during a one-year follow-up [15,16]. GA provides better access to the teeth and enhances isolation, which is necessary for a successful fissure sealant therapy. Therefore, under GA, their success rate increased to 95.17%, as reported in the current study, and 92.8% in the study by Amin et al. [9]. It should be considered that 43% of the failed sealant treatments, in this study, needed restoration due to dental caries. Some of these (new caries) might have started from interproximal surfaces and secondarily resulted in the failure of the fissure sealants.

The failure rate of restorative treatments, in the current study, was relatively lower as compared to that of the previous studies. The failure rates of posterior and anterior composite restoration in the current study were 9.96% and 9.44%, respectively. Previous studies reported failure rates of 13.8% [17] and 28.3% [10] for anterior composites and 6.6% [17] and 9.7% [10] for posterior composites. Regarding amalgam posterior fillings, the failure rate was lower (5.33%); however, previous studies have reported failure rates ranging from 7.8% [17,18] to 21% [12] for amalgam restorations. The difference could be explained by the improvements in restorative materials as well as in the technique.

Finally, the failure rate of SSC was 5.26% in the current study. Some previous studies reported lower failure rates for SSC (1% [10,12], 1.5% [18], 1.9% [17] to 2.8% [9], 3% [19], 3.8% [20], and 4.5% [2]), whereas, others had higher failure rates (7.2% [11] and 8% [12]). Although previous studies revealed that SSC has higher success rates compared to the other restorations [21,22,23], in this study, there was little difference between the failure rates of amalgam restorations and SSC. Moreover, both had lower failures compared to that of posterior composite restorations. Since most of the failures of restorations are due to new carious lesions, SSC might play a protective role in such a case. A recent systematic review revealed the superiority of SSCs in regard to the posterior primary teeth [24]. On the other hand, the failure rate of SSC in this study might be due to a failure in the pulp therapy rather than restoration. Similar to the previous studies, the reason for failure was not determined in the current study.

The very low failure rate of pulp therapies in this study was in agreement with the findings of previous studies. The failure rates of pulpectomy and pulpotomy were 2.90% and 3.03%, respectively. In addition, previous studies reported low failure rates of 0.2% [17] and 0.8% [18] for pulpectomy and 1.1% [18], 1.2% [17], 2% [19], and 2.9% [2] for pulpotomies; however, the study conducted by Drummond et al. [11] reported a higher failure rate (15.4%) for pulpotomies performed under GA. This difference might be due to the fact that they performed a retrospective evaluation of hospital documents.

In comparison to the study by Lin et al. [25], who assessed the factors influencing the development of caries after GA, there was no difference in the failure rate between boys and girls in this study, except regarding anterior composite restorations. The results also demonstrated no association of treatment failure with the educational level of the mothers; however, some studies have demonstrated the effect of awareness of the mothers regarding the dental health status of their children [26,27,28].

Although fluoride therapy following restorative treatments could reduce the bacterial load and virulence, which might increase the treatment success rate [29], the results of the current study failed to depict the effect of participation in regular post-operative fluoride therapy sessions on the failure rate of the treatments. On the contrary, Biria et al. [18] demonstrated that regular fluoride therapy is associated with a higher success rate of dental treatments performed under GA. In addition, Sheller et al. [13] reported that attendance to regular active follow-ups decreases the need to repeat dental treatments under GA.

One of the limitations of this study was that the reason for treatment failure was not assessed. It was not clear whether the reported failure was due to the patient’s factors, dental material, or the operator. To this end, the relatively long follow-up in this study, as compared to previous ones, provides an overall view for the clinicians to know what to expect in each treatment.

There are some possible risks of bias in this study. The first is that those who did not participate in the follow-up session might have had higher or lower failure rates for the different treatments. The parents might bring their child to the follow-up if they felt the treatment had failed. In addition, due to the retrospective method of this study, there might be some mistakes in the documents and the patients’ data. To that end, the authors tried to reduce this error by performing a meticulous assessment of the documents, not including patients with incomplete documents, and inspecting the retrospective data from the follow-up sessions.

Another limitation of this study was the clinical assessment of the restorative and endodontic treatments. Radiolucencies, resorptions, and interproximal caries could be assessed more accurately using radiography. Radiographic examination was not approved by the ethical committee as we did not intend to retreat the failures; instead, the parents were merely informed of the results and referred to a specialist. However, similar to the current study, the clinical assessment of the success factors has been used by previous authors [12,17,18]. Others studies reported failure and success through the assessment of the treatment need without explaining the details of the clinical examination process [9,10].

## 4. Materials and Methods

### 4.1. Study Design and Population

This study was performed as a multicenter retrospective cohort study. The study protocol was approved by the local university’s ethical committee (date: 13 December 2013). The patients included children who had received dental treatment under general anesthesia during the years 2010–2013 in two private hospitals in Babol in northern Iran. The treatments were provided by five pediatric dentistry specialists. The inclusion and exclusion criteria are listed in Table 4.

The patients were recalled for the follow-up session. In this session, the patients were examined and they received tooth polishing and fluoride therapy as well as instructions for further treatments, if necessary. All examinations were performed by a trained dentist.

The demographic data including the patient’s age (at the time of GA) and gender as well as the parents’ educational level was recorded. Then, the history regarding the patient’s participation in regular fluoride therapies (every 3–4 months) during the first year after GA and retreatments or extraction of the treated teeth were obtained from the patients’ documents.

### 4.2. Clinical Examination

In this study, only the primary teeth were evaluated. The evaluations included only a clinical examination with no radiographic assessment. The simplified oral health index (OHI-S), modified for primary dentition [30], was used for the assessment of oral hygiene. This index is similar to the original OHI-S [31] and is performed on the six predefined primary teeth, including the upper right second deciduous molar, the upper right central deciduous incisor, the upper left second deciduous molar, the lower right second deciduous molar, the lower left central deciduous incisor, and the lower left second deciduous molar. The validity of the OHI-S has been demonstrated in previous studies [30,31]. The OHI-S has two components, namely, the simplified debris index (DI-S) and the simplified calculus index (CI-S). Each of them indicates the amount of debris and the calculus on the predefined teeth surfaces using a scale of 0–3 (0 indicates the absence of debris or calculus, whereas 3 indicates more than two thirds of the assessed tooth surface are covered with debris or calculus). The total score is between 0 and 6.

The number of primary teeth with decay (*d*), missing (*m*), and filling (*f*) was recorded and the dmft index of each patient was calculated.

Regarding the patients who previously had had follow-ups after their treatment under GA, any previous extractions that were performed due to extensive caries or infection as well as any retreatments were considered as treatment failure and recorded. In addition, the present need for retreatment was recorded as failure based on the specific criteria determined to assess the failure of each treatment modality. For teeth that received more than one treatment (for example, pulp therapy and restoration), each treatment was independently evaluated based on its own failure criteria. The fissure sealants, which were completely or partially lost, were counted as a failure. Regarding the assessment of amalgam and composite restorations, three parts of the criteria developed by Cvar and Ryge [32], including marginal integrity, anatomical shape, and secondary caries, were used. If one of the following signs were found during clinical examination, the restoration was considered as a failure: a gap between the tooth and the restoration with exposed dentin, loss of the restoration form with exposed dentin, and secondary caries along the margins [18]. Regarding stainless steel crowns (SSC), the observation of gingivitis, crown mobility, or loss were considered as the failure. Finally, oral soft tissue and periodontium around the endodontically treated teeth were examined and for pulp therapies, the presence of dental abscess, fistula, or inflammation was recorded as a failure.

## 5. Statistical Analysis

The percentage of failure was calculated for each treatment by dividing the number of failures by the total number of treatments. The comparison of the treatment failures was performed using the Chi square test. A regression model was applied in order to assess the effects of the influencing factors (age, gender, parents’ education, oral hygiene, follow-up time, fluoride therapy, and dmft index) on the risk of failure. Multiple regression analysis was used to evaluate and predict the failure rate (outcome) based on the influencing factors (predictors). The statistical analysis was performed using SPSS software v.18 (SPSS Inc., Chicago, IL, USA) with a significance level of 0.05.

## 6. Conclusions

Within the limitations of this retrospective study, it could be concluded that the overall failure rate of dental treatments that are performed on pediatric patients under GA was 6.59%, wherein pulp therapies had the lowest and composite restorations had the highest rate of failure.

There was no association between the patients’ age, gender, oral hygiene, follow-up time, fluoride therapy, dmft index, and the parents’ education with the failure rate of the treatments, except that girls had a higher rate of failure in anterior composite restorations and age affected the failure of SSC and fissure sealant therapies.

## Figures and Tables

**Table 1:** Oral health status of the patients at the time of follow-up.

Index	N	Range	Mean (SD)
OHI-S	114	1–6	2.67 (1.27)
decay (*d*)	114	0–4	1.21 (1.07)
missing (*m*)	114	0–4	0.69 (0.87)
filling (*f*)	114	3–16	8.88 (2.51)
dmf	114	5–19	10.78 (2.70)
OHI-S: simplified oral health index; SD: standard deviation			

**Table 2:** Failure rate of each treatment modality.

Treatment	No. Patients	Females	Age ^¥^ (Months) ^§^		Follow-up (Months) ^§^		No. of Treated Teeth	No. of Failed Treated Teeth (Failure Rate %)
			Mean	SD	Mean	SD		
Fissure sealant	58	17	37.69	11.39	33.22	15.85	145	7 (4.83)
Posterior composite	88	29	37.28	10.94	33.41	15.40	261	26 (9.69)
Anterior composite	109	39	37.24	10.67	33.48	15.25	466	44 (9.44)
**Total composite**	114	40	37.17	10.75	33.97	15.59	727	70 (9.63)
Amalgam	81	29	38.38	10.39	35.95	14.84	169	9 (5.33)
SSC	65	20	37.49	11.04	29.15	14.20	114	6 (5.26)
Pulpotomy	85	32	38.82	10.91	33.88	15.86	198	6 (3.03)
Pulpectomy	99	34	37.15	10.48	33.91	15.11	241	7 (2.90)
**Total pulp therapy**	113	39	37.32	10.68	34.07	15.62	439	13 (2.96)
**Total**	114	40	37.17	10.75	33.97	15.59	1594	105 (6.59)

^¥^ age at the time of treatment under general anesthesia; ^§^ calculated for patients who had each treatment; SSC: Stainless Steel Crown; SD: Standard Deviation.

**Table 3:** Failure rate of each treatment modality based on follow-up time.

Treatment	7–12 Months			13–24 Months			25–36 Months			37–48 Months			49–60 Months			60–66 Months		
	No. of Treated Teeth	No. of Failed Treated Teeth	Failure Rate (%)	No. of Treated Teeth	No. of Failed Treated Teeth	Failure Rate (%)	No. of Treated Teeth	No. of Failed Treated Teeth	Failure Rate (%)	No. of Treated Teeth	No. of Failed Treated Teeth	Failure Rate (%)	No. of Treated Teeth	No. of Failed Treated Teeth	Failure Rate (%)	No. of Treated Teeth	No. of Failed Treated Teeth	Failure Rate (%)
Fissure sealant	14	0	0	26	0	0	36	4	11.11	31	0	0	24	1	4.167	14	2	14.29
Posterior composite	30	1	3.33	49	2	4.08	75	5	6.67	66	11	16.67	26	2	7.69	15	5	33.33
Anterior composite	42	2	4.76	80	6	7.50	126	13	10.32	129	13	10.08	52	6	11.54	37	4	10.81
**Total composite**	72	3	4.17	129	8	6.20	201	18	8.95	195	24	12.31	78	8	10.26	52	9	17.31
Amalgam	10	0	0	25	1	4.00	51	2	3.92	35	1	2.86	34	2	5.88	14	3	21.43
SSC	9	0	0	37	0	0	34	2	5.88	27	3	11.11	5	1	20.00	2	0	0
Pulpotomy	17	0	0	41	0	0	54	3	5.56	44	2	4.54	27	1	3.70	15	0	0
Pulpectomy	21	2	9.52	48	1	2.08	60	1	1.67	69	1	1.45	30	1	3.33	13	1	7.69
**Total pulp therapy**	38	2	5.26	89	1	1.12	114	4	3.51	113	3	2.65	57	2	3.51	28	1	3.57
**Total**	253	10	3.95	524	19	3.62	751	52	6.92	709	58	8.18	333	24	7.21	190	25	13.16

**Table 4:** Inclusion and exclusion criteria.

**Inclusion Criteria**
Pediatric patients who received dental treatment under general anesthesia Generally healthy patients Reason for anesthetizing was lack of cooperation or dental phobia More than six months post-operative follow-up Participating in the follow-up session Treated tooth was present at the time of follow-up (except those extracted due to treatment failure)
**Exclusion Criteria**
Pediatric patients who had physical or mental disabilities Systemic diseases Lack of cooperation during follow-up session Incomplete documents Teeth that were exfoliated
